# Effect of Timely Availability of TTR-Stabilizing Therapy on Diagnosis, Therapy, and Clinical Outcomes in ATTR-CM

**DOI:** 10.3390/jcm13175291

**Published:** 2024-09-06

**Authors:** Stephan Dobner, Sara Zarro, Fabian Wieser, Mohammad Kassar, Bashir Alaour, Sebastian Wiedemann, Adam Bakula, Federico Caobelli, Stefan Stortecky, Christoph Gräni, Lukas Hunziker, Benedikt Bernhard

**Affiliations:** 1 Department of Cardiology, Inselspital, Bern University Hospital, University of Bern, 3010 Bern, Switzerland; sara.zarro@usz.ch (S.Z.); fabian.wieser@ik.me (F.W.); mohammad.kassar@insel.ch (M.K.); bashir.alaour@kcl.ac.uk (B.A.); sebastian.wiedemann@hin.ch (S.W.); adam.bakula@insel.ch (A.B.); stefan.stortecky@insel.ch (S.S.); christoph.graeni@insel.ch (C.G.); lukas.hunziker@insel.ch (L.H.); benni.bernhard@web.de (B.B.); 2 3rd Medical Department of Cardiology and Intensive Care Medicine, Clinic Ottakring (Former Wilhelminenhospital), 1160 Vienna, Austria; 3 British Heart Foundation Centre for Research Excellence, King’s College London, London SE1 9NH, UK; 4 Department of Nuclear Medicine, Inselspital, Bern University Hospital, University of Bern, 3010 Bern, Switzerland; federico.caobelli@insel.ch

**Keywords:** cardiac amyloidosis, transthyretin, tafamidis

## Abstract

**Highlights:**

**What is known?**

- Tafamidis, a transthyretin stabilizer, reduces cardiovascular morbidity and mortality in patients with transthyretin amyloid cardiomyopathy (ATTR-CM).

**What is new?**

- Availability of tafamidis increases diagnostic efficacy reducing time-to-diagnosis and time-to-therapy initiation.
- Timely diagnosis and availability of therapy allow therapy initiation and optimization of supportive therapies at earlier disease stages and translate into improved clinical outcomes by reducing heart failure hospitalizations and all-cause mortality.

**What is next?**

- Future studies are needed to examine whether faster initiation of TTR-targeting therapies improves long-term morbidity and mortality and to identify which patients benefit most from early therapy.

**Abstract:**

**Background**: Tafamidis reduces cardiovascular morbidity and mortality in transthyretin amyloid cardiomyopathy (ATTR-CM), yet availability and access to therapy vary. **Objective**: To determine how availability and access to tafamidis impact time-to-diagnosis, time-to-therapy, and cardiovascular outcomes in ATTR-CM. **Methods**: Ninety-one consecutive ATTR-CM (~97% wt-TTR) patients diagnosed between June 2019 and June 2021 were evaluated for tafamidis. Access to therapy was regulated by compassionate use [n(CU) = 42] prior to, and insurance [n(IA) = 49] after regulatory approval. **Results**: Tafamidis was started in 37/42 (88.1%), and 39/49 (79.6%) patients, respectively. At diagnosis, ATTR-CM disease stage (≤stage 2: 88.2% vs. 90.9%, *p* = 0.92) was similar between groups. Timely access (after tafamidis approval) reduced the median time from first presentation to diagnosis from 6.2 (IQR: 1.3–28.9) to 2.4 (0.7–21.7) months, and from first presentation to therapy from 24.4 (10.7–46.8) to 11.8 (6.4–32.4) months. While RV function significantly worsened between diagnosis and therapy initiation in CU patients diagnosed before tafamidis approval (S’-velocity 10.0 ± 2.2 to 9.2 ± 2.2 cm/s; *p* = 0.018; TAPSE 17.3 ± 4.7 to 15.7 ± 3.9 mm, *p* = 0.008), it remained unchanged in IA patients (S’-velocity 9.6 ± 2.6 to 9.4 ± 2.3 cm/s; *p* = 0.83; TAPSE 15.6 ± 4.2 to 16.3 ± 3.1 mm, *p* = 0.45). After a median follow-up of 42.3 and 24.9 months in CU and IA patients, respectively, timely availability was associated with a reduction in annual heart failure hospitalizations (0.40 vs. 0.16 per patient, *p* < 0.001) and improved MACE-free survival (HR = 0.51; 95%CI: 0.26–1.00; *p* = 0.051). Timely diagnosis (<12-months) prolonged MACE-free survival (HR = 0.424; 95%CI: 0.22–0.81; *p* = 0.004), and reduced HFH (HR = 0.40; 95%CI: 0.19–0.81); *p* = 0.011) and all-cause mortality (HR = 0.29; 95%CI: 0.11–0.74); *p* = 0.009). **Conclusions**: Availability of tafamidis improves diagnostic efficacy in ATTR-CM patients. Timely diagnosis and initiation of therapy reduces adverse cardiovascular events.

## 1. Introduction

The emergence of effective therapeutic options for amyloid transthyretin cardiomyopathy (ATTR-CM) has contributed to increased awareness and recognition of the disease [1,2]. Tafamidis and acoramidis, two transthyretin (TTR) stabilizers, have successfully completed phase 3 clinical trials, significantly improving cardiovascular outcomes in ATTR-CM patients [1,3]. The results of the HELIOS-B clinical trial, which tested the effects of vutrisiran, a small interfering RNA therapeutic, on all-cause mortality and recurrent cardiovascular events, are expected shortly (at ESC 2024), with a recent press release suggesting a significant reduction in primary outcome events [4]. To date, tafamidis remains the only TTR-targeting therapy widely used in clinical practice. TTR stabilization with tafamidis improves survival and quality of life, and reduces cardiovascular-related hospitalizations and functional decline [1]. Yet, the availability of tafamidis varies, not least due to the high cost associated with this therapy [5]. Recognition of disease prevalence [6] and advanced cardiac diagnostics [7] enable the detection of the disease at earlier stages with more favorable outcomes [2] adding to the debate on the optimal timing of therapy initiation and the ideal candidacy for targeted ATTR-CM therapy [2].

In 2019, prior to local regulatory drug approval in Europe, early tafamidis access for patients previously diagnosed with ATTR-CM was granted by the manufacturer through an expanded access program (compassionate use, CU). For patients diagnosed after regulatory drug approval in April 2020, tafamidis was more readily available, yet subject to insurance coverage of therapy costs (insurance access, IA).

The primary aim of the current study was to evaluate the effect of tafamidis availability and wider access to treatment on the diagnostic efficacy (time from first presentation to diagnosis) and therapy initiation (time from first presentation to therapy) in patients with ATTR-CM. Our secondary endpoint was to assess how these changes and delays in diagnosis and therapy influence cardiovascular outcomes in ATTR-CM patients.

## 2. Methods

### 2.1. Study Population

Consecutive patients referred to the Cardiac Amyloidosis Clinic at the Department of Cardiology, Bern University Hospital, Inselspital, Bern, Switzerland, and diagnosed with ATTR-CM between June 2019 and June 2021, were prospectively enrolled in the Bern Amyloidosis registry (B-CARE) (NCT04776824) upon written, informed consent. Baseline clinical and follow-up data were recorded using standardized, electronic case report forms and entered into a dedicated online database at Bern University Hospital. The study design was approved by the local ethics committee (KEK: 2021-00135) and conducted in accordance with the Declaration of Helsinki.

### 2.2. ATTR-CM Diagnosis

ATTR-CM was diagnosed non-invasively if bone scintigraphy detected moderate or severe myocardial ^99m^Tc labelled 3,3-diphosphono-1,2-propanodicarboxylic acid (^99m^Tc-DPD) tracer uptake (Perugini ≥ 2) [8] after exclusion of light chain (AL) amyloidosis by a gammopathy panel consisting of serum gel electrophoresis, serum immunofixation, and serum free light chain assay [9]. If tissue biopsies confirmed TTR amyloid deposits, a biopsy-based diagnosis was made, with cardiac imaging [echocardiography or cardiac magnetic resonance imaging (CMR)] required to confirm cardiac involvement in patients with extracardiac amyloid deposits.

Genetic testing was performed as previously described [10] after written, informed consent. Peripheral blood samples were used for DNA extraction, and testing was performed using Sanger sequencing of all exons and exon–intron boundaries of the transthyretin gene.

### 2.3. Time of First Presentation, Time of Diagnosis

Time of first presentation was assessed using electronic patient records and defined as first documentation of symptomatic heart failure (HF) and unexplained left ventricular (LV) wall thickness >12 mm. In patients diagnosed non-invasively, time of diagnosis was defined as the date of the diagnostic bone scintigraphy or CMR. For patients undergoing biopsy, the date of diagnosis was the date of myocardial biopsy or the date of cardiac imaging (TTE or CMR) confirming cardiac involvement in patients with a TTR-positive extracardiac biopsy.

### 2.4. Echocardiography

Transthoracic echocardiography was performed at first presentation, 6–12 monthly during follow-up, and prior to initiating tafamidis therapy. The most recent echocardiography exam at or prior to diagnosis was used for baseline assessment.

### 2.5. Criteria for TTR-Stabilizing Therapy with Tafamidis

Qualification for tafamidis therapy was based on the ATTR-ACT study [1]. Patients presenting before April 2021 were evaluated for treatment eligibility through the tafamidis expanded access program (compassionate use, CU) provided by the drug manufacturer. Patients evaluated after regulatory drug approval in April 2021 were evaluated for tafamidis therapy and cost coverage through their health insurance (insurance access, IA). While the CU program extended to patients presenting with NYHA III at the time of therapy commencement, stricter prescription criteria applied after tafamidis regulatory drug approval April 2021 for patients evaluated for IA (NYHA I-II at therapy initiation, prior heart failure hospitalization or requirement for diuretic, NT-proBNP > 600 pg/mL, GFR > 25 mL/min/1.73 m^2^, 6 min walk test >100 m, life expectancy >2 years).

### 2.6. Follow-Up and Clinical Endpoints

Clinical follow-up data were obtained through standardized interviews during clinic visits, documentation from referring physicians, and hospital discharge summaries. Current mortality data were provided by the Swiss Federal Statistical Office. Adverse events were systematically collected and adjudicated by two independent board-certified cardiologists. The endpoints of the study were diagnostic efficacy (time from first presentation to diagnosis), time to therapy (time from first presentation to therapy), and major adverse cardiovascular events (MACEs), which included all-cause mortality and heart failure hospitalizations (HFH).

### 2.7. Statistical Analysis

Statistical analysis was performed with IBM SPSS Statistics 25 (IBM Corp., Armon, NY, USA) and R software version 4.1.3 (R Foundation for Statistical Computing, Vienna, Austria). Baseline characteristics are presented as numeric frequencies (percentages), mean ± standard deviation or as median with the 25th and 75th percentiles whenever appropriate. The study cohort was dichotomized into patients evaluated for therapy through the CU program before regulatory drug approval and those evaluated for therapy through IA after drug approval. Group characteristics were compared by chi-square tests or Fisher’s exact tests for categorical variables, by unpaired *t*-tests for continuous normally distributed variables, and by Mann–Whitney U tests for highly skewed variables. Changes in numeric variables between the timepoint of diagnosis and therapy initiation were evaluated by paired *t*-tests. Cox proportional hazards regression analysis was conducted to investigate univariate associations with the combined endpoint of HFH and all-cause death. Time-to-event curves were plotted using the Kaplan–Meier method for (1) a composite endpoint of HFH and all-cause mortality, (2) HFH, and (3) all-cause mortality, and the corresponding *p*-values for log-rank tests were provided. The cumulative incidence of the combined endpoint and its components was described by the cumulative annualized event rates and compared among groups by independent *t*-tests. Recurrent heart failure hospitalizations were visualized using a cumulative incidence function, with all-cause death considered as a competing event. A two-sided *p*-value was <0.05 was considered statistically significant.

## 3. Results

**Figure 1:**
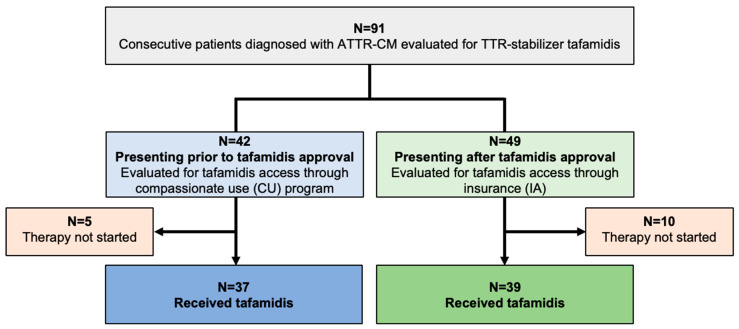
Study CONSORT flow chart. Abbreviations: ATTR-CM—transthyretin amyloid cardiomyopathy, TTR—transthyretin, CU—compassionate use, IA—insurance access.

In total, 91 consecutive patients diagnosed with ATTR-CM were evaluated for TTR-stabilizing therapy with tafamidis (Study Flow Chart, Figure 1). A share of 37 of 42 patients (88.1%) diagnosed prior to tafamidis approval fulfilled the criteria to commence therapy and tafamidis was thus provided through the drug manufacturer (CU cohort). Of the 49 patients presenting after tafamidis approval (IA cohort), 39 (79.6%) qualified for therapy, and were started on tafamidis after approval of insurance cost coverage was obtained.

### 3.1. Baseline Characteristics

Detailed baseline characteristics at the time of diagnosis for the CU and IA cohorts, respectively, are shown in Table 1. The mean age of participants in the CU and IA groups was 76.3 ± 6.4 and 77.3 ± 6 years, respectively, with predominantly patients of male sex in both groups (95.2% and 93.9%, respectively, *p* > 0.99). Other than a more prevalent history of atrial fibrillation (59.5% vs. 38.8%; *p* = 0.048) and carpal tunnel syndrome (31.0% vs. 12.2%; *p* = 0.029) in the CU group, baseline characteristics and medical history were comparable between the groups. At diagnosis, both groups had similar levels of eGFR (59.4 ± 18.2 vs. 57.2 ± 34.5 mL/min) and NT-proBNP [median (IQR) 1806 (967–3104) vs. 1678 (979–4226) pg/mL], and ATTR-CM disease stage was comparable between groups (*p* = 0.92). A non-invasive scintigraphy-based diagnosis of ATTR-CM was made in 88.1% of patients in the CU cohort, and this proportion increased to 98% in the IA cohort, which presented after tafamidis approval. A similar proportion of patients in both groups presented with strong (89.2% vs. 89.6%) and moderate (10.8% vs. 10.4%) ^99^Tc-DPD-tracer uptake, scintigraphically indicative of similar disease stages in the CU and IA cohort, respectively. Echocardiographic evaluation revealed comparable biventricular function in the low–normal range in both groups [53.87 ± 11.57% (CU) vs. 52.0 ± 10.83% (IA)]; however, maximum LV wall thickness was significantly higher in the CU cohort (18.8 ± 3.3 vs. 16.9 ± 3.2 mm; *p* = 0.035).

### 3.2. Time to Diagnosis and Time to Therapy

**Figure 2:**
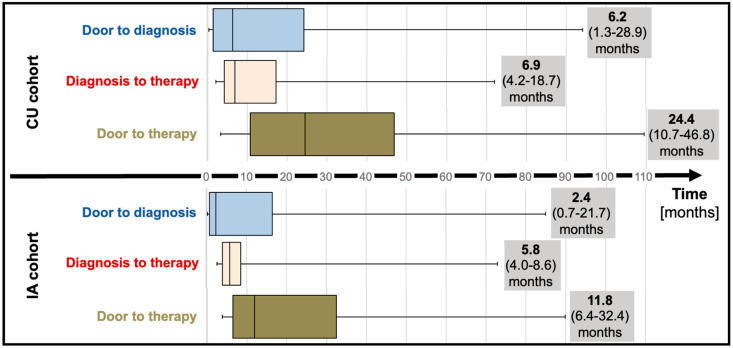
Time to diagnosis (median and interquartile range) and time to therapy (median and interquartile range) before (compassionate use) and after tafamidis approval (insurance access). Abbreviations: CU—compassionate use, IA—insurance access.

Median “door to diagnosis” time (time from first presentation to diagnosis) was 6.2 months (IQR; 1.3 to 28.9) for CU patients and numerically decreased to 2.4 months (IQR; 0.7 to 21.7, *p* = 0.20) for the IA cohort (Table 1; Figure 2). After diagnosis, referral of patients to our reference center and administrative requirements to commence therapy (consultation at the reference center, electronic application for therapy for the CU cohort, letter to request insurance approval for the IA cohort) delayed therapy initiation by a median of 6.9 months (IQR; 4.2 to 18.7) and 5.8 months (IQR; 4.8 to 8.6) for CU and IA cohorts, respectively. Taken together, “door to therapy” time (time from first presentation to therapy initiation) added up to 24.4 months (IQR; 10.7 to 46.8) in CU patients. In IA patients, a reduced “door to therapy” of 11.8 months (IQR; 6.4 to 32.4, *p* = 0.13) was observed.

### 3.3. Temporal Changes in Clinical ATTR-CM Disease Stage and Echocardiographic Characteristics from Time of Diagnosis to Therapy Initiation

Clinical, biochemical, and echocardiographic data at diagnosis and initiation of tafamidis therapy were available for 31 of 37 (83.8%) CU patients and 37 of 39 (94.9%) IA patients, respectively (Table 2). ATTR-CM disease stage, cardiorenal biomarkers, and NYHA functional class did not significantly change in either group. While structural and functional echocardiographic parameters remained stable in the IA cohort, RV function measured by RV S’-velocity (from 10.0 ± 2.2 to 9.2 ± 2.2; *p* = 0.018) and tricuspid annular plane systolic excursion (TAPSE) (from 17.3 ± 4.7 to 15.7 ± 3.9; *p* = 0.008) significantly decreased in CU patients (Table 2).

### 3.4. Clinical Outcomes after ATTR-CM Diagnosis

**Figure 3:**
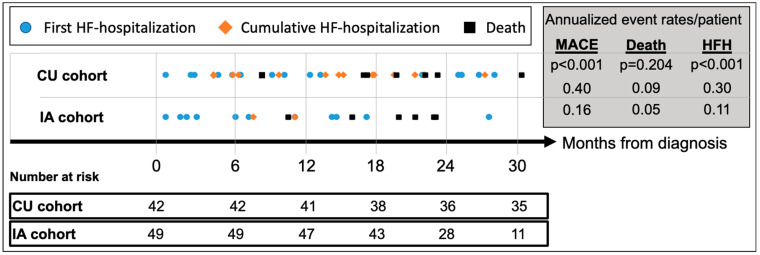
Cumulative incidence of adverse events since diagnosis of ATTR-CM. Abbreviations: HFH—heart failure hospitalizations, MACE—major adverse cardiovascular event (=HFH or death), CU—compassionate use, IA—insurance access.

Median follow-up for the CU and IA patients was 42.3 (IQR 35.2–49.0) and 24.9 (IQR 20.1–29.8) months, respectively (Table 3). MACE (HFH or death) occurred in 24 (57.1%) CU patients and 13 (26.5%) IA patients, respectively, translating to an annualized recurrent MACE rate of 0.40/patient (95%CI_0.30–0.51) in the CU cohort vs. 0.16/patient (95%CI_0.09–0.25) in the IA cohort (*p* < 0.001) (Figure 3). Compared to CU patients, annualized repeat HFH rates per patient were significantly lower in the IA cohort [CU: 0.30 (95%CI_0.23–0.41) vs. IA: 0.11 (95%CI_0.06–0.18); *p* < 0.001], while annualized mortality rates per patient did not differ [CU: 0.09 (95%CI 0.05–0.15) vs. IA: 0.05 (95%CI 0.02–0.11); *p* = 0.20].

After commencement of tafamidis therapy, the composite endpoint of all-cause mortality or HFH occurred in 17 (45.9%) CU patients and 6 (17.8%) IA patients, respectively, with an annualized recurrence rate of 0.38/patient (95%CI 0.28–0.52) in the CU cohort vs. 0.17/patient (95%CI 0.08–0.28) in the IA cohort (*p* = 0.012) (Table 3). Compared to CU patients, annualized repeat HFH rates per patient were significantly lower in the IA cohort [CU: 0.30 (95%CI 0.22–0.44) vs. IA: 0.12 (95%CI 0.05–0.23); *p* < 0.011] while annualized mortality rates per patient again did not differ [CU: 0.09 (95%CI 0.04–0.17) vs. IA: 0.05 (95%CI 0.01–0.15); *p* = 0.40].

**Figure 4:**
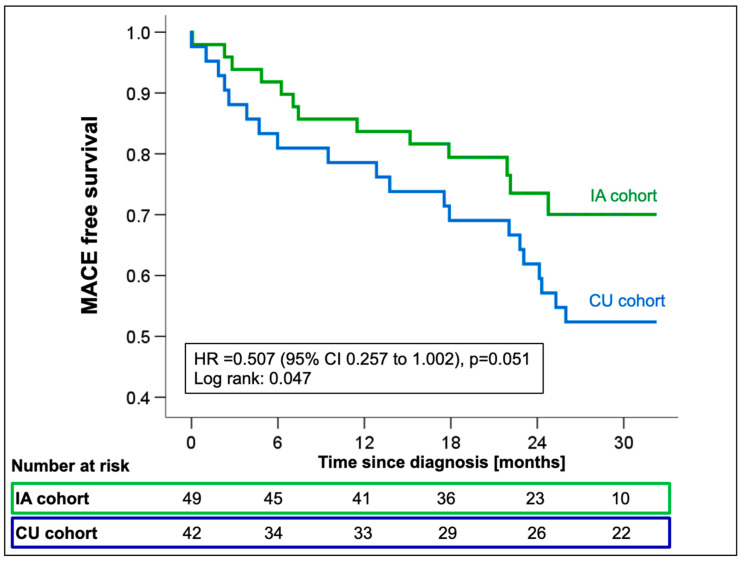
Kaplan–Meier estimates for first MACE from the time of ATTR-CM diagnosis stratified by the availability of tafamidis [compassionate use (CU) vs. insurance access (IA)]. Abbreviations: HR—hazard ratio, MACE—major adverse cardiovascular events.

Cox regression analysis showed a significant reduction in the incidence of all-cause mortality or first HFH in the IA cohort with a marginally non-significant hazard ratio (HR 0.51; 95%CI 0.26–1.00; *p* = 0.051, Figure 4) compared to CU patients.

When looking at all-cause mortality and HFH individually, the risk reduction resulted mainly from a trend towards a reduction in HFH (HR 0.50; 95%CI 0.23–1.05; *p* = 0.067), while mortality rates were comparable between groups (HR 0.58; 95%CI 0.22–1.55; *p* = 0.28, Supplemental Figure S1). The combined endpoint of first MACE was univariately associated with creatinine (HR 1.01; 95%CI 1.00–1.02; *p* = 0.015), eGFR (HR 0.97; 95%CI 0.95–0.99; *p* = 0.004), and log-transformed NT-proBNP (HR 5.64; 95%CI 2.1–15.2; *p* = 0.001, Supplemental Table S1) measured at the time of diagnosis.

### 3.5. Effect of Timely Diagnosis on Clinical Outcomes in ATTR-CM Patients

To test the predictive value of time to diagnosis on clinical outcomes in our cohort, we stratified patients by time from first presentation to diagnosis with a cut-off at 12 months (group 1: diagnosed < 12 months vs. group 2: >12 months from presentation to diagnosis). Patients diagnosed within 12 months had significantly lower LV maximal wall thickness (16.8 ± 2.1 vs. 18.8 ± 3.7 mm; *p* = 0.014), lower LV mass (147.3 ± 32.9 vs. 178.7 ± 49.3 g/m^2^; *p* = 0.003), and more preserved kidney function (eGFR: 63 ± 17 vs. 55 ± 17.6 mL/min; *p* = 0.033, creatinine: 104 ± 29.6 vs. 117.3 ± 35 mmol/L; *p* = 0.034) at the time of diagnosis (Table 4). 

**Figure 5:**
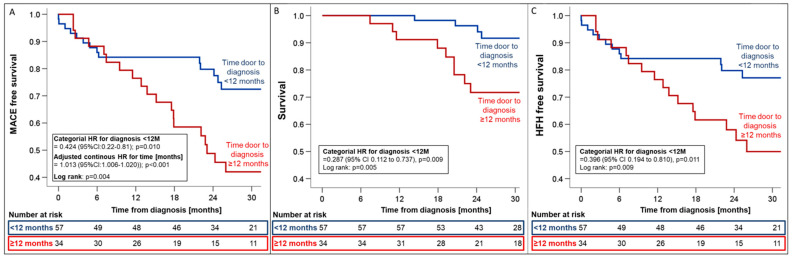
Kaplan–Meier estimates for first MACE (**A**), all-cause mortality (**B**), and HFH (**C**) from the time of ATTR-CM diagnosis stratified by the time from first presentation to diagnosis (<12 months vs. >12 months). Hazard ratios were adjusted for variables with univariate association with the combined endpoint (i.e., eGFR and NT-proBNP). Abbreviations: HR—hazard ratio, MACE—major adverse cardiovascular events.

Timely diagnosis within 12 months was associated with relative risk reductions of 57.4%, 71.3%, and 60.4% for MACE (HR 0.42; 95%CI_0.22–0.81, *p* = 0.01, Figure 5), all-cause mortality (HR 0.29; 95%CI_0.11–0.73; *p* = 0.009, Figure 5), and HFH (HR 0.40; 95%CI_0.19–0.81; *p* = 0.011, Figure 5), respectively. 

In a multivariate Cox regression model, log-transformed NT-proBNP (HRadjusted 6.47; 95%CI_1.82–23.03; *p* = 0.004) and delayed time to diagnosis (HRadjusted 1.01; 95%CI_1.01–1.02; *p* = 0.002) remained independent predictors of adverse outcomes, with each additional month of delayed diagnosis increasing the relative risk of MACE by 14% (Supplemental Table S1).

### 3.6. Effect of Atrial Fibrillation on Clinical Outcomes in ATTR-CM Patients

To evaluate the effect of atrial fibrillation on clinical outcomes in our cohort, we stratified patients by history of atrial fibrillation at the time of diagnosis. A history of atrial fibrillation significantly increased the risk for first MACE (HR 2.62; 95%CI: 1.33–5.17, *p* = 0.005, Supplemental Figures S5 and S6), first HFH (HR 3.12; 95%CI: 1.43–6.82, *p* = 0.004, Supplemental Figure S5), and cumulative HFHs (Supplemental Figure S6). All-cause mortality (HR 1.61; 95%CI: 0.64–4.04, *p* = 0.86, Supplemental Figure S5) was comparable irrespective of a history of atrial fibrillation.

## 4. Discussion

The availability of TTR-targeting therapeutics has changed the outlook for patients with ATTR-CM, significantly reducing heart failure hospitalizations and all-cause mortality, while preserving exercise capacity and quality of life [1]. To gain a deeper understanding of the impact of tafamidis availability on clinical practice and patient outcomes, we compared time to diagnosis, time-to-therapy initiation, and clinical outcomes of patients diagnosed prior to tafamidis market approval whose access to tafamidis was regulated by the manufacturers’ expanded access program (CU cohort) to patients diagnosed after market approval (IA cohort) with timely access provided via insurance coverage. Availability of tafamidis was associated with reductions in both time to diagnosis and time to therapy in the IA cohort. Increased disease awareness amongst physicians, familiarity with diagnostic modalities, and familiarity with therapeutic options all likely contributed to the reduction in median time to diagnosis from 6.2 (IQR; 1.3 to 28.9) in the CU cohort to 2.4 months (IQR; 0.7 to 21.7, *p* = 0.20) in the IA cohort. Together, faster diagnosis and more timely referrals to our tertiary center for therapy evaluation reduced the time from first presentation to therapy initiation from 24.4 months (IQR 10.7–46.8) in CU patients to 11.8 months (IQR 6.4–32.4, *p* = 0.13) in IA patients. While these reductions were not statistically significant due to large time variabilities, potentially indicative of varying levels of disease awareness by treating physicians and the small sample size, the high efficacy of 18-month treatment with tafamidis, with a number needed to treat (NNT) of 7.5 patients to prevent a HFH or death over 18 months as demonstrated in ATTR-ACT [1], suggests that these median reductions of 3.8 (from first presentation to diagnosis) and 12.6 months (from first presentation to therapy) are clinically meaningful.

As seen in the observational long-term extension study of ATTR-ACT [11], early access to tafamidis improved outcomes in our patients primarily by reducing HFHs (Supplemental Figure S1). To elucidate potential mechanisms underlying improved outcomes with early adoption of tafamidis, we compared clinical, structural, and biochemical disease characteristics at diagnosis and therapy initiation, respectively. In patients diagnosed with ATTR-CM prior to market approval of tafamidis, structural and biochemical disease progression was observed as RV function declined and NT-proBNP levels increased. Both parameters—NT-proBNP as a component of the most widely used staging classification, and TAPSE as measure of RV function—have previously been identified as prognostic markers for mortality in ATTR-CM [12,13]. Stabilization of cardiac structural changes, thereby preserving RV function and steadying NT-proBNP levels (Table 2), were benefits seen in IA patients from early therapeutic intervention, and these may contribute to an amelioration of clinical disease progression.

Significantly improved clinical outcomes with a reduction in both HFH and all-cause mortality were observed when patients were stratified by time to diagnosis (Figure 5). Notably, these improvements were consistent, irrespective of the availability of tafamidis at the time of diagnosis. As for NT-proBNP (HR_adjusted_ 6.47; 95%CI_1.82–23.03; *p* = 0.004), uni- and multivariate Cox-regression analyses confirmed delayed time to diagnosis (HR_adjusted_ = 1.01; 95%CI_1.01–1.02; *p* = 0.002) to be an independent predictor of MACE (Supplemental Table S1). These findings are likely attributable to a multitude of factors, including diagnosis at an earlier disease stage with less advanced structural disease and a lower prevalence of atrial fibrillation when event rates are likely lower [2], timely access to tafamidis, and also optimization of supportive medical [14,15,16], interventional [17,18], and device therapies [19]. While randomized evidence for heart failure therapy in ATTR-CM remains elusive, the adverse effects of a beta-blockade, particularly in more advanced disease, when chronotropic incompetence limits cardiac output in patients with a fixed stroke volume, have repeatedly been described [20]. Thus, in line with expert opinion [21], discontinuation of beta-blockers is typically recommended for our patients at the time of ATTR-CM diagnosis. Likewise, without evidence of a beneficial effect of neurohormonal blockers even in patients with reduced ejection fraction [15], cessation of these drugs may help to prevent adverse events, e.g., from orthostatic hypotension or progressive kidney dysfunction caused by lower-than-required blood pressure targets. In line with the observation of an increased risk for MACE and HFH in ATTR-CM patients with a history of atrial fibrillation at diagnosis, we hypothesize that an aggressive and early pursuit of sinus rhythm in patients developing atrial fibrillation is yet another likely contributor to improved outcomes after the diagnosis of ATTR-CM [22], as is the choice of a physiologic pacing modality (CRT or LBBAP) [19], to prevent disease deteriorations more likely to be seen in ATTR-CM with high RV-only pacing burden.

Increased awareness and diagnosis at earlier ATTR-CM disease stages have called the optimal timing of therapy initiation into question [2], particularly in light of the high economic burden of costly TTR-targeting therapies [5]. Yet, ATTR-CM remains a progressive disease, without reliable methods to monitor disease progression and to allow for timely commencement of therapy. With promising new therapies that may allow reversal of amyloid deposition on the horizon [23] but not yet realized, our data suggest that with delays from first presentation to diagnosis still common, early therapeutic intervention should be sought to prevent adverse clinical outcomes in ATTR-CM patients.

## 5. Limitations

The current investigation was a retrospective, unblinded, observational study and subject to multiple biases (e.g., disease awareness by physicians, referral bias, selection bias, varying follow-up time), which change over the course of the study. The study was conducted at a single tertiary reference center in Switzerland. After market approval, tafamidis became widely available to Swiss patients within 6 months. As the rate of implementation and mode of tafamidis prescription vary regionally, the improvements in diagnostic efficacy observed in our cohort may not be generalizable. The study cohort was overwhelmingly male, and suffering from wt-ATTR-CM, reflecting current screening recommendations for ATTR-CM. Extrapolation of study findings to female patients with ATTR-CM or those suffering from h-ATTR-CM may therefore not be warranted. Lastly, as enrolment into the compassionate use program was limited to 9 months, the study’s sample size was limited, increasing the likelihood of random error and chance findings.

## 6. Conclusions

Increased availability and access to tafamidis shortened time-to-diagnosis and time-to-therapy initiation for symptomatic wt-ATTR-CM patients. Timely diagnosis and early commencement of therapy were associated with a reduction in adverse cardiovascular events, providing an opportunity for treating physicians to improve patient outcomes.

## Figures and Tables

**Table 1:** Patient characteristics at time of ATTR-CM diagnosis.

	Compassionate Use (CU)	Facilitated Access through Insurance (IA)	*p*-Value
**N**	42	49	N/A
**Patient characteristics**			
Sex [male] *n (%)*	40 (95.2)	46 (93.9)	>0.99
Age [years] *mean ± SD*	76.3 ± 6.4	77.3 ± 6	0.45
**Medical history *n (%)***			
Arterial hypertension	25 (59.5)	29 (59.2)	0.97
Diabetes mellitus	7 (16.7)	10 (20.4)	0.65
Coronary artery disease	16 (38.1)	13 (26.5)	0.24
Atrial fibrillation	25 (59.5)	19 (38.8)	**0.048**
PPM implanted	3 (7.1)	4 (8.2)	>0.99
NYHA-class			
I	15 (35.7)	9 (18.4)	0.35
II	21 (50.0)	29 (59.2)	
III	6 (14.3)	9 (18.4)	
IV	0	2 (4.1)	
**ATTR-characteristics *n (%)***			
History of carpal tunnel syndrome	13 (31)	6 (12.2)	**0.029**
History of polyneuropathy	5 (11.9)	4 (8.2)	0.73
History of spinal stenosis	3 (7.1)	7 (14.3)	0.33
History of valvular cardiopathy	7 (16.7)	7 (14.3)	0.75
**Biomarkers**			
hs-Troponin T [ng/L] *mean ± SD*	55.4 ± 33.8	57.2 ± 34.5	0.82
Creatinine [mmol/L] *mean ± SD*	112 ± 35.5	106.3 ± 29	0.42
eGFR [ml/min] *mean ± SD*	59.4 ± 18.2	60.8 ± 17	0.73
NT-proBNP [pg/mL] *median (IQR)*	1806 (967–3104)	1678 (979–4226)	0.81
ATTR-CM stage I	20 (58.8)	26 (59.1)	0.92
ATTR-CM stage II	10 (29.4)	14 (31.8)	
ATTR-CM stage III	4 (11.8)	4 (9.1)	
**Diagnostics *n (%)***			
DPD scintigraphy performed	37 (88.1)	48 (98)	N/A
Perugini II	4 (10.8)	5 (10.4)	>0.99
Perugini III	33 (89.2)	43 (89.6)	
Gammopathy panel sampled	41 (97.6)	47 (95.9)	N/A
Abnormalities found	7 (17.1)	6 (12.8)	0.57
Genetic testing performed	39 (92.8)	44 (89.8)	N/A
Wild type TTR	38 (97.4)	43 (97.7)	>0.99
**Medication *n (%)***			
ACE-inhibitor	11 (26.2)	9 (18.4)	0.37
AT1-antagonist	14 (33.3)	17 (34.7)	0.89
Sacubitril/Valsartan	2 (4.8)	0	0.22
Betablocker	21 (50)	24 (49)	0.92
Mineralocorticoid recepter antagonist	9 (21.4)	12 (24.5)	0.73
Diuretic	25 (59.5)	33 (67.3)	0.44
Loop diuretic dose [mg]	17.5 ± 14.5	11.7 ± 8.7	0.10
TTR-stabilizing therapy initiated	37 (88.1)	39 (79.6)	0.28
**Echocardiography** *mean ± SD*			
LVEF [%]	53.9 ± 11.6	52 ± 10.8	0.45
LV GLS [%]	−9.1 ± 5.5	−10.3 ± 4.3	0.35
LV EDV Index [mL/m^2^]	40.7 ± 14.6	49.4 ± 18.1	0.12
LV Mass Index [g/m^2^]	172.3 ± 52.8	162.3 ± 39.7	0.38
LV maximal wall thickness [mm]	18.8 ± 3.3	16.9 ± 3.2	**0.04**
RV DTI S-Wave Velocity [cm/s]	9.7 ± 2.2	10.4 ± 3	0.34
TAPSE [mm]	16.6 ± 4.9	16.6 ± 5.4	0.96
LAVi [mL/m^2^]	43.7 ± 11.5	48.1 ± 11.2	0.14
**Timing [months]** *median (IQR)*			
Door to diagnosis	6.2 (1.3 to 28.9)	2.4 (0.7 to 21.7)	0.20
Diagnosis to therapy	6.9 (4.2 to 18.7)	5.8 (4.0 to 8.6)	0.41
Door to therapy	24.4 (10.7 to 46.8)	11.8 (6.4 to 32.4)	0.13
Potential earliest therapy start to actual start	4.5 (3.6 to 6.4)	5.8 (4.0 to 8.6)	**0.034**

**Abbreviations**: ACE—angiotensin converting enzyme, AT1—angiotensin 1, DPD—99mTc-DPD Technetium^99^-3,3-diphosphono-1,2-propanodicarboxylic acid, DTI—doppler tissue imaging, EDV—end diastolic volume, eGFR—estimated glomerular filtration rate, GLS—global longitudinal strain, hs—high sensitivity, LAVi—left atrial volume index, LVEF—left ventricular ejection fraction, NT-proBNP—N-terminal pro hormone of brain natriuretic peptide, NYHA—New York Heart Association functional class, PPM—permanent pacemaker, TAPSE—Tricuspid annular plane systolic excursion, TTR—Transthyretin. ATTR-CM disease stage: Stage I = NT- proBNP ≤ 3000 ng/L and GFR ≥ 45 mL/min, Stage III = NT-proBNP > 3000 ng/L and GFR < 45 mL/min; the remaining patients are classified as Stage II.

**Table 2:** Temporal changes in clinical characteristics, ATTR-CM disease stage, and echocardiographic parameters from diagnosis to therapy initiation.

	Compassionate Use (CU)			Facilitated Access through Insurance (IA)		
N (on Therapy/with Complete FU Data)	37/31			39/37		
	At Diagnosis	At Therapy Initiation	*p*-Value	At Diagnosis	At Therapy Initiation	*p*-Value
**NHYA-class *n (%)***						
I	13 (41.9)	12 (38.7)	0.75	8 (21.6)	11 (29.7)	0.22
II	15 (48.4)	14 (45.2)		21 (56.8)	24 (64.9)	
III	3 (9.7)	5 (16.1)		7 (18.9)	2 (5.4)	
IV	0	0		1 (2.7)	0	
**Biomarkers**						
Creatinine [mmol/L] *mean ± SD*	110.5 ± 36.57	114.1 ± 38.9	0.20	108.4 ± 30	111 ± 26	0.42
eGFR [mL/min] *mean ± SD*	60.7 ± 18.6	58.3 ± 18.8	0.14	60.2 ± 17.6	58.3 ± 15.3	0.32
NTproBNP [pg/mL] *median (IQR)*	2117 ± 1316	2720 ± 1944	0.059	2983 ± 3522	2206 ± 1977	0.072
hs-Troponin T [ng/L] *mean ± SD*	51.6 ± 28.4	59 ± 40.3	0.32	56.1 ± 27	51.1 ± 18.1	0.32
**ATTR-CM disease stage**						
Stage I	17 (58.6)	20 (54.1)	0.85	21 (58.3)	22 (59.5)	0.88
Stage II	10 (34.5)	13 (35.1)		12 (33.3)	13 (35.1)	
Stage III	2 (6.9)	4 (10.8)		3 (8.3)	2 (5.4)	
**Echocardiography**						
LVEF [%]	53.8 ± 11.8	52.5 ± 11.5	0.22	50.1 ± 11.9	49.2 ± 14.2	0.66
LV GLS [%]	−9.0 ± 6.1	−10.7 ± 3.7	0.23	−9.5 ± 2.7	−9.3 ± 3.1	0.78
LV EDV Index [mL/m^2^]	38.4 ± 15.1	38.2 ± 14.4	0.78	45.4 ± 19.3	40.1 ± 13.9	0.30
LV Mass Index [g/m^2^]	161.6 ± 42.6	161.1 ± 41.1	0.94	157.8 ± 33.3	159.5 ± 37.5	0.82
LV maximal wall thickness diastole [mm]	18.8 ± 3.6	18.6 ± 3.7	0.60	15.9 ± 2.4	16.9 ± 2.4	0.28
RV DTI S-Wave Velocity [cm/s]	10.0 ± 2.2	9.2 ± 2.2	**0.018**	9.6 ± 2.6	9.4 ± 2.3	0.83
TAPSE [mm]	17.3 ± 4.7	15.7 ± 3.9	**0.008**	15.6 ± 4.2	16.3 ± 3.1	0.45
LAVi [ml/m^2^]	43.6 ± 11.8	43.9 ± 11.3	0.91	50.5 ± 16.4	49.6 ± 16.7	0.63

**Table 3:** Clinical outcomes after ATTR-CM diagnosis and commencement of tafamidis therapy.

	Compassionate Use (CU)	Facilitated Access through Insurance (IA)	*p*-Value
**Outcome since diagnosis**			
N	42	49	
Observation time [months] median (IQR)	42.3 (35.2 to 49.0)	24.9 (20.1 to 29.8)	
Any MACE n (%)	24 (57.1)	13 (26.5)	
Cumulative annualized MACE rate (95%CI)	0.40 (0.30–0.51)	0.16 (0.09–0.25)	**<0.001**
Cumulative annualized rate of HFH (95%CI)	0.30 (0.23–0.41)	0.11 (0.06–0.18)	**<0.001**
Death n (%)	13 (30.9)	6 (12.2)	
Annualized mortality rate (95%CI)	0.09 (0.05–0.15)	0.05 (0.02–0.11)	0.20
**N (with therapy)**	**37**	**39**	
**Patients with event between time of diagnosis and therapy initiation**			
HFH n (%)	4 (10.8)	3 (7.7)	
Death n (%)	0	0	
**Outcomes after tafamidis initiation**			
Observation time [months] *median (IQR)*	33.4 (30.1 to 35.9)	17.8 (14.5 to 21.9)	
Any MACE *n (%)*	17 (45.9)	6 (15.4)	
Cumulative annualized MACE rate (95%CI)	0.38 (0.28–0.52)	0.17 (0.08–0.28)	**0.012**
Cumulative annualized rate of HFH (95%CI)	0.30 (0.22–0.44)	0.12 (0.05–0.23)	**0.011**
Death *n (%)*	8 (21.6)	3 (7.7)	
Annualized death rate (95%CI)	0.09 (0.04–0.17)	0.05 (0.01–0.15)	0.40

**Abbreviations**: HFH—heart failure hospitalizations, MACE—major adverse cardiovascular events.

**Table 4:** Effect of time to diagnosis on ATTR-CM disease stage at diagnosis.

	Time to Diagnosis in ATTR-CM Patients		
	Diagnosis < 12 M	Diagnosis > 12 M	
	N = 57	N = 34	*p*-Value
**NYHA-class *n (%)***			
I	15 (26.3)	9 (26.4)	0.94
II	32 (56.1)	18 (52.9)	
III	9 (15.8)	6 (17.6)	
IV	1 (2)	1 (3)	
**Biomarkers**			
Creatinine [mmol/L] *mean ± SD*	104 ± 29.6	117.3 ± 35	**0.034**
eGFR [mL/min] *mean ± SD*	63 ± 17	55 ± 17.6	**0.033**
NTproBNP [pg/mL] *median (IQR)*	1697 (967–3956)	1770 (1012–2887)	0.57
hs-Troponin T [ng/L] *mean ± SD*	55.3 ± 34.6	58.1 ± 33.2	0.37
**ATTR-CM disease stage**			
Stage I	27 (55.1)	19 (65.5)	0.071
Stage II	19 (38.8)	5 (17.2)	
Stage III	3 (6.1)	5 (17.2)	
**Echocardiography**			
LVEF [%]	54.5 ± 11	51.9 ± 11.5	0.27
LV GLS [%]	−9.5 ± 5.7	−10.1 ± 3.1	0.34
LV EDV Index [mL/m^2^]	45.1 ± 15.9	45.5 ± 18.8	0.47
LV Mass Index [g/m^2^]	147.3 ± 32.9	178.7 ± 49.3	**0.003**
LV maximal wall thickness [mm]	16.8 ± 2.1	18.8 ± 3.7	**0.014**
RV DTI S-Wave Velocity [cm/s]	10.4 ± 2.8	9.5 ± 2.4	0.12
TAPSE [mm]	17 ± 5.3	16 ± 4.7	0.23
LAVi [mL/m^2^]	45.1 ± 15.8	45.5 ± 18.8	0.44

## Data Availability

The data presented in this study are available on request from the institution. The data are not publicly available due to privacy/ethical considerations.

## Supplementary Materials

The following supporting information can be downloaded at: https://www.mdpi.com/article/10.3390/jcm13175291/s1, Figure S1. Kaplan–Meier estimates for all-cause mortality and time to first HFH since the time of diagnosis stratified by the availability of tafamidis [compassionate use (CU) vs. insurance access (IA)]; Figure S2. Kaplan–Meier estimates for first MACE (A), all-cause mortality (B), and HFH (C) stratified by tafamidis therapy; Figure S3. Kaplan–Meier estimates for first MACE (A), all-cause mortality (B), and HFH (C) from the time of ATTR-CM diagnosis for patients treated with tafamidis stratified by the time from first presentation to diagnosis (<12 months vs. >12 months); Figure S4. Cumulative incidence function for repeat HFH from the time of ATTR-CM diagnosis for patients treated with tafamidis stratified by the time from first presentation to diagnosis (<12 months vs. >12 months); Figure S5. Kaplan–Meier estimates for first MACE (A), all-cause mortality (B), and HFH (C) from the time of ATTR-CM diagnosis for patients with and without a history of or concomitant atrial fibrillation at the time of ATTR-CM diagnosis; Figure S6. Cumulative incidence function for repeat HFH from the time of ATTR-CM diagnosis for patients with and without a history of concomitant atrial fibrillation at the time of ATTR-CM diagnosis; Table S1. Associations of clinical characteristics at the time of diagnosis with the combined endpoint by Cox regression.

## Abbreviations

ATTR-CM	Cardiac Transthyretin Amyloid Cardiomyopathy
GFR	Glomerular Filtration Rate
HFH	Heart failure hospitalization
HR	Hazard ratio
LVEF	Left Ventricular Ejection Fraction
MACE	major adverse cardiovascular events
NT-proBNP	N-terminal pro hormone of Brain Natriuretic Peptide
NYHA	New York Heart Association functional class
TAPSE	Tricuspid annular plane systolic excursion
TTR	Transthyretin
^99m^Tc-DPD	Technetium^99^-3,3-diphosphono-1,2-propanodicarboxylic acid
wt-TTR	wild-type tranythyretin
