# Supplementary material for: Effect of Timely Availability of TTR-Stabilizing Therapy on Diagnosis, Therapy, and Clinical Outcomes in ATTR-CM
Source: J Clin Med. 2024 Sep 6;13(17):5291. doi: 10.3390/jcm13175291 (PMC11396384; doi:10.3390/jcm13175291)

SUPPLEMENTAL MATERIAL

Dobner et al.

Effect of timely availability of TTR-stabilizing therapy on diagnosis, therapy, and clinical outcomes in ATTR-CM

ClinicalTrials.gov Identifier: NCT04776824

Supplemental Material

Supplementary Table S1. Associations of clinical characteristics at the time of diagnosis to the combined endpoint by Cox regression

	**Univariate associations**		**Multivariate model**	
	**Hazard ratio (95%CI)**	**p value**	**Hazard ratio (95%CI)**	**p value**
**Patient characteristics**				
Gender (female vs. male)	2.187 (95% CI 0.667 to 7.167)	0.20		
Age [years]	1.048 (95% CI 0.987 to 1.113)	0.12		
NHYA-class (III or IV vs. I or II)	1.454 (95% CI 0.676 to 3.125)	0.34		
**Biomarkers**				
Creatinine [mmol/l]	1.011 (95% CI 1.002 to 1.020)	**0.015**		
eGFR [ml/min]	0.970 (95% CI 0.950 to 0.990)	**0.004**	0.989 (95% CI 0.963 to 1.016)	0.42
NTproBNP [Log pg/ml]	5.64 (95% CI 2.087 to 15.23)	**0.001**	6.47 (95% CI 1.818 to 23.03)	**0.004**
hs-Troponin T [ng/l] *mean ± SD*	1.007 (95% CI 0.999 to 1.016)	0.07		
**Echocardiography**				
LVEF [%]	0.979 (95% CI 0.949 to 1.010)	0.18		
LV GLS [%]	1.023 (95% CI 0.953 to 1.098)	0.53		
LV Mass Index [g/m^2]	1.000 (95% CI 0.992 to 1.007)	0.93		
RV DTI S-Wave Velocity [cm/s]	0.945 (95% CI 0.815 to 1.096)	0.45		
TAPSE [mm]	1.001 (95% CI 0.925 to 1.083)	0.99		
LAVi [ml/m^2]	1.012 (95% CI 0.978 to 1.048)	0.50		
**Timing** [months]				
First presentation to diagnosis	1.013 (95% CI 1.006 to 1.020)	**<0.001**	1.014 (95% CI 1.005 to 1.023)	**0.002**
Diagnosis to therapy	1.050 (95% CI 0.983 to 1.012)	0.19		
First presentation to therapy	1.009 (95% CI 0.997 to 1.021)	0.15		
**CU vs. IA**	1.972 (95% CI 0.998 to 3.896)	0.051		

Supplemental figure S1. Kaplan Meier estimates for all-cause mortality and time-to-first HFH since the time of diagnosis stratified by the availability of tafamidis [compassionate use (CU) vs. insurance access (IA)].

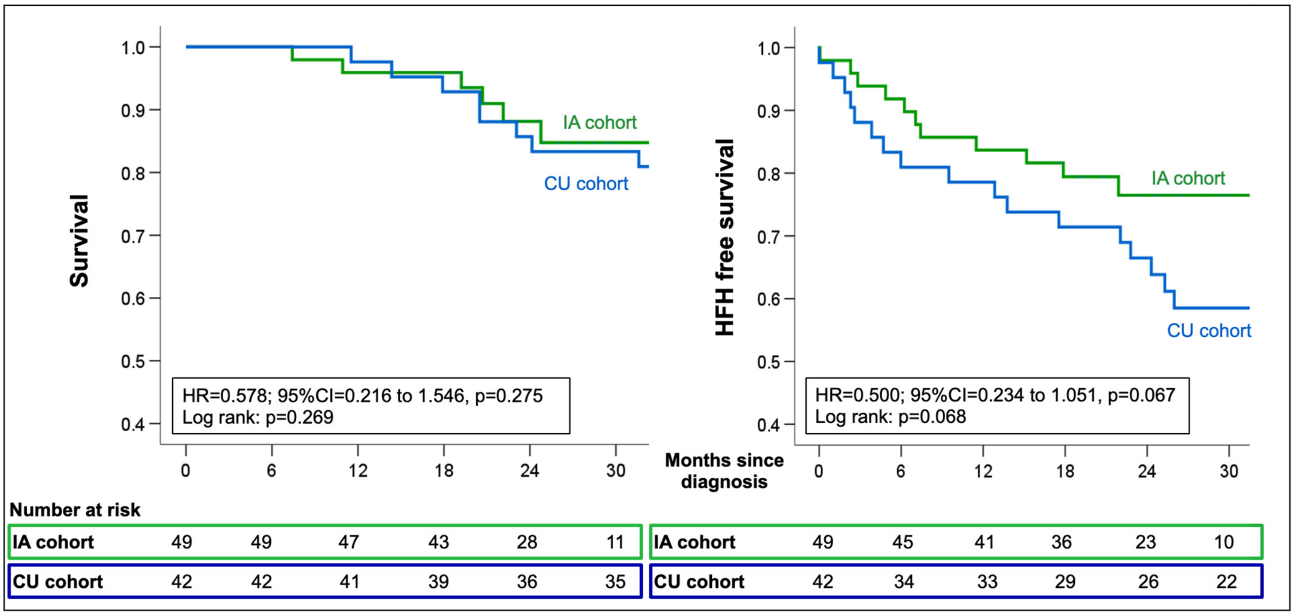

Supplemental figure S2. Kaplan Meier estimates for first MACE (A), all-cause mortality (B) and HFH (C) stratified by tafamidis therapy.

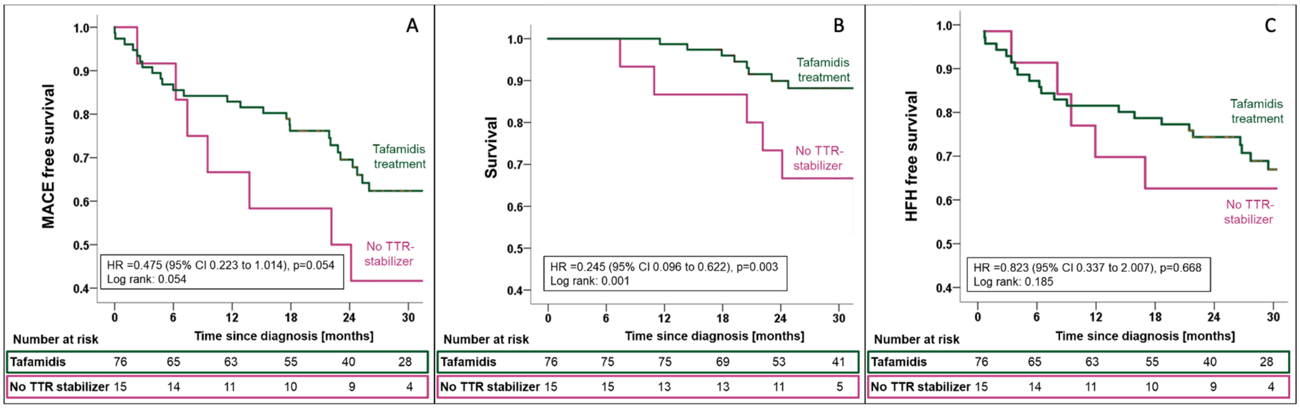

Supplemental figure S3. Kaplan Meier estimates for first MACE (A), all-cause mortality (B) and HFH (C) from the time of ATTR-CM diagnosis for patients treated with tafamidis stratified by the time from first presentation to diagnosis (<12months vs. >12 months).

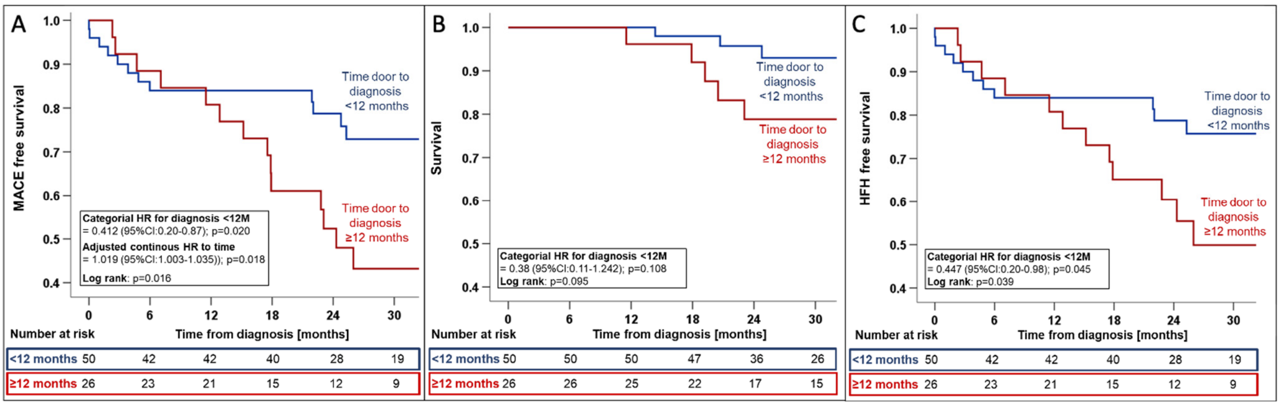

Hazard ratios were adjusted for variables with univariate association to the combined endpoint (i.e. eGFR and NT-proBNP).

Supplemental figure S4. Cumulative incidence function for repeat HFH from the time of ATTR-CM diagnosis for patients treated with tafamidis stratified by the time from first presentation to diagnosis (<12months vs. >12 months).

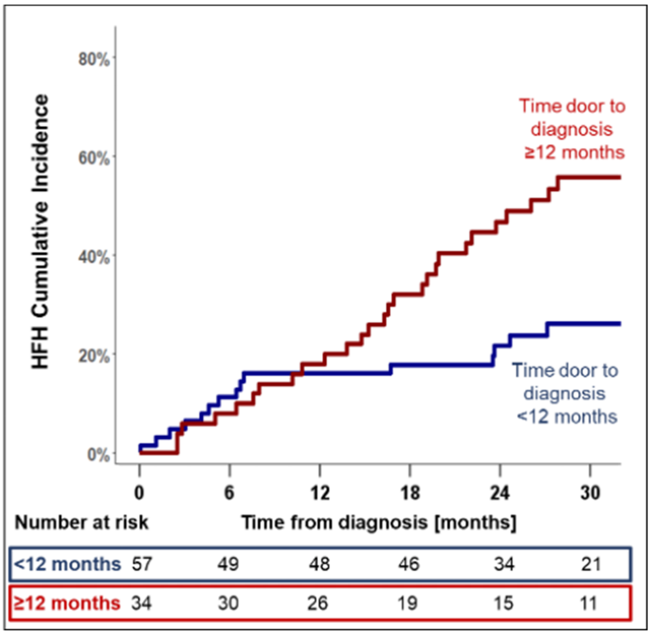

Supplemental figure S5. Kaplan Meier estimates for first MACE (A), all-cause mortality (B) and HFH (C) from the time of ATTR-CM diagnosis for patients with and without a history of or concomitant atrial fibrillation at the time of ATTR-CM diagnosis.

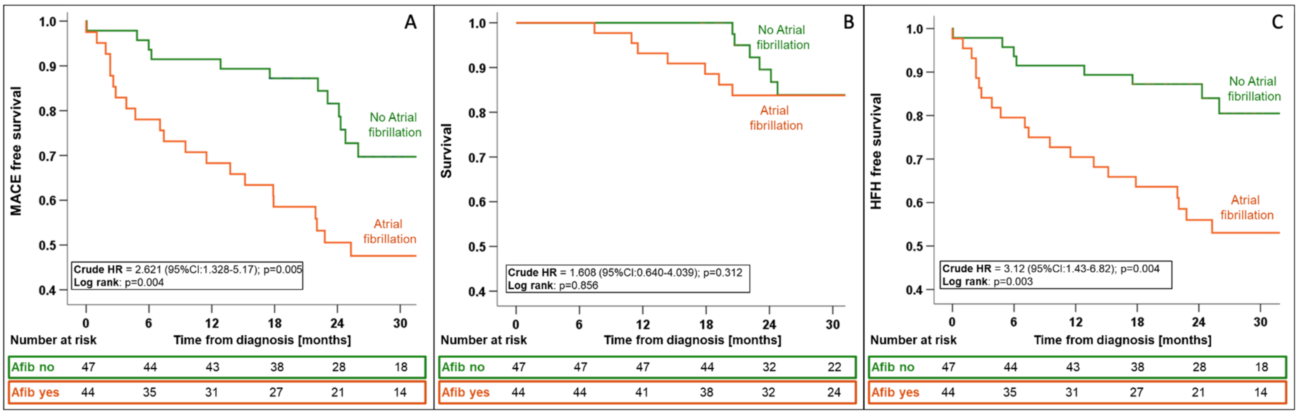

Supplemental figure S6. Cumulative incidence function for repeat HFH from the time of ATTR-CM diagnosis for patients with and without a history of or concomitant atrial fibrillation at the time of ATTR-CM diagnosis.

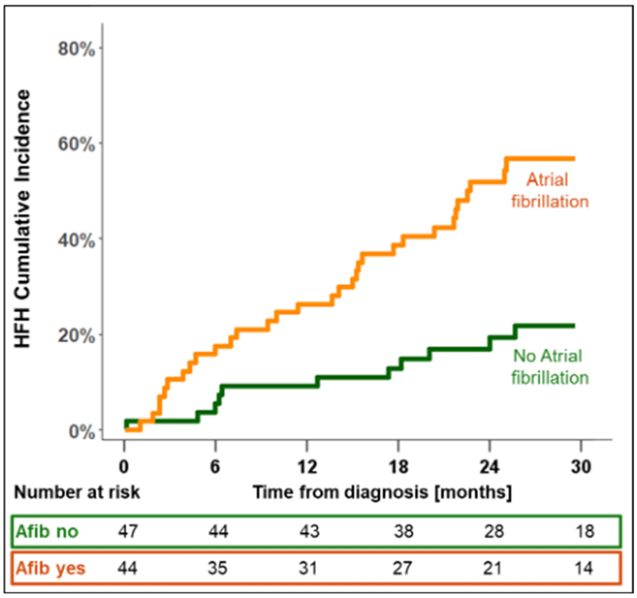

Supplement: Supplementary file 1 [file jcm-13-05291-s001.zip › jcm-3185224-supplementary.pdf]
